# Integrated Investigation Approach for Solid Waste Landfill Hazards—A Case Study of Two Decommissioned Industrial Sites

**DOI:** 10.3390/toxics13100807

**Published:** 2025-09-23

**Authors:** Xiaoyu Zhang, Aijing Yin, Yuanyuan Lu, Zhewei Hu, Li Sun, Wenbing Ji, Qi Li, Caiyi Zhao, Yanhong Feng, Lingya Kong, Rongrong Ying

**Affiliations:** Nanjing Institute of Environmental Sciences, Ministry of Ecology and Environment, Nanjing 210042, China

**Keywords:** solid waste landfill, geophysical detection, drilling, visualized geological modeling

## Abstract

Historical chemical production sites often harbor irregularly distributed solid waste landfills, posing significant environmental risks. Traditional drilling methods, while accurate, are inefficient for comprehensive characterization due to high costs and spatial limitations. This study aims to develop an integrated geophysical drilling approach to accurately delineate the spatial distribution and volume of landfilled solid waste (predominantly organic pollutants) at two decommissioned chemical plant sites (total area: 8954 m^2^). Methods: We combined (1) geophysical surveys (transient electromagnetic (TEM, 50 profiles, 2936 points), high-density resistivity (HDR, 2 profiles, 192 points), and ground-penetrating radar (GPR, 22 profiles, 1072.1 m)) and (2) systematic drilling verification (136 boreholes, ≤10 m × 10 m density). Anomalies were interpreted through integrating geophysical responses, historical records, and borehole validation. Spatial modeling was conducted using Kriging interpolation in EVS software. The results show that (1) the anomalies exhibited a “sparse multi-point distribution” across zones A2 (primary waste concentration), A4, and A6, which were differentiated into solid waste, foundations, contaminated soil, voids, and cracks; (2) drilling confirmed solid waste at nine locations (A2: “multi-point, small-quantity” residues; A6: contaminated clay layers with garbage) with irregular thicknesses (0.2–1.3 m); (3) TEM identified diagnostic medium–high-resistivity anomalies (e.g., 28–37 m in A4L3), while GPR detected 17 shallow anomalies (only one validated as waste); and (4) the total waste volume was quantified as 266.9 m^3^. The methodology reduced the field effort by ∼35% versus drilling-only approaches, resolved geophysical limitations (e.g., HDR’s volume effect overestimating the thickness), and provided a validated framework for efficient characterization of complex historical landfills.

## 1. Introduction

Historical chemical industrial sites represent significant environmental liabilities, with undocumented solid waste landfills posing severe risks to groundwater and soil integrity across China’s industrialized regions [1,2,3]. Traditional drilling methods involve direct contact investigation, resulting in accuracy and high precision. However, the use of grid or randomly distributed sampling points leads to a limited number of drilling locations, making it challenging to comprehensively and precisely locate the landfill position and range [4,5,6,7]. The investigation process is lengthy, lacks real-time monitoring, and is costly. Compared with traditional drilling sampling methods, geophysical detection methods offer advantages such as non-destructive in situ assessment, high spatial sampling density, rapid data collection, and lower costs [8,9]. Geophysical methods are increasingly applied in environmental pollution investigations, including investigations of landfill boundaries, leachate leakage contamination, organic pollutants, underground solid waste landfill, and contaminated site rehabilitation, as well as monitoring of effectiveness evaluation processes. Critically, historical chemical industry sites in China (e.g., Jiangsu Province) face undocumented waste disposal with groundwater contamination risks, demanding efficient characterization methods.

By utilizing geophysical detection methods, pollutants are detected based on their physical properties compared with the surrounding media, with multiple geophysical techniques used in combination to complement and verify each other in identifying the distribution range of pollutants [10,11,12,13,14]. Currently, geophysical methods for detecting organic pollution include high-density electrical resistivity, transient electromagnetic, ground-penetrating radar, and multi-frequency electromagnetic detection methods [15,16,17,18]. While these methods have their own advantages and disadvantages, the high-density electrical resistivity method is notable for its quick construction, cost-effectiveness, large dataset, high resolution, strong interference resistance, and intuitive imaging; the transient electromagnetic method offers advantages such as low detection costs, high efficiency, minimal susceptibility to terrain effects, and the ability to penetrate high-resistivity cover layers, albeit with limitations in shallow-depth detection; the multi-frequency electromagnetic detection method demonstrates high operational efficiency, fieldwork convenience, and cost-effectiveness, and can swiftly delineate anomalous zones based on electrical and magnetic properties, yet it possesses limited detection depth.

In investigating solid waste landfill sites, the main objectives include determining the scope, depth, types of pollutants, degree of contamination, and contaminant dispersion extent. Investigating the scope, depth, and contamination level involves a combination of drilling as the primary method and geophysical detection as a supplementary method. In recent years, geophysical methods have been increasingly applied to investigations in solid waste landfill sites, expanding from their original use in various engineering fields. According to a literature research, geophysical methods commonly applied in solid waste landfill site investigations include electrical resistivity, electromagnetic, ground-penetrating radar, and magnetic methods. Among these, the electrical resistivity method is particularly effective given favorable conditions at solid waste landfill sites and its proven success in qualitative and semi-quantitative assessments of pollution extents. However, due to inherent accuracy limitations, existing methods fail to resolve thin waste layers (<0.5 m) and lack standardized integration protocols for industrial sites [19], creating a critical methodological gap for historical chemical plant investigations. In addition, while empirical parameters have been used to determine the pollution range based on electrical detection results, there is a lack of comprehensive, reliable correlations between pollutant types, concentrations, and geoelectric characteristics, hindering quantitative pollution analysis. To address these challenges, combining the accuracy and high precision of drilling results with the non-destructive, rapid, and broad coverage capabilities of geophysical methods can offer more comprehensive and accurate investigation results.

This study focuses on investigating solid waste landfill at two decommissioned industrial sites in Huaihe River Terrace, Jiangsu (total area: 8954 m^2^) using geophysical methods for non-destructive detection on a broad scale (“surface”) and drilling methods for precise surveys (“points”). This combined approach aims to (1) establish an integrated TEM-HDR-GPR workflow for thin-layer waste detection (0.2–1.3 m), (2) develop resistivity–concentration correlations for organic pollutants (phenyl chlorosilanes), and (3) propose standardized procedures for historical site characterization. In addition, a three-dimensional visualization model of the landfill layers was constructed using Earth Volumetric Studio (EVS) software (v2020.5.2, USA), exploring the spatial distribution characteristics of solid waste landfill and proposing practical approaches and methods for solid waste landfill investigations.

## 2. Materials and Methods

**Figure 1:**
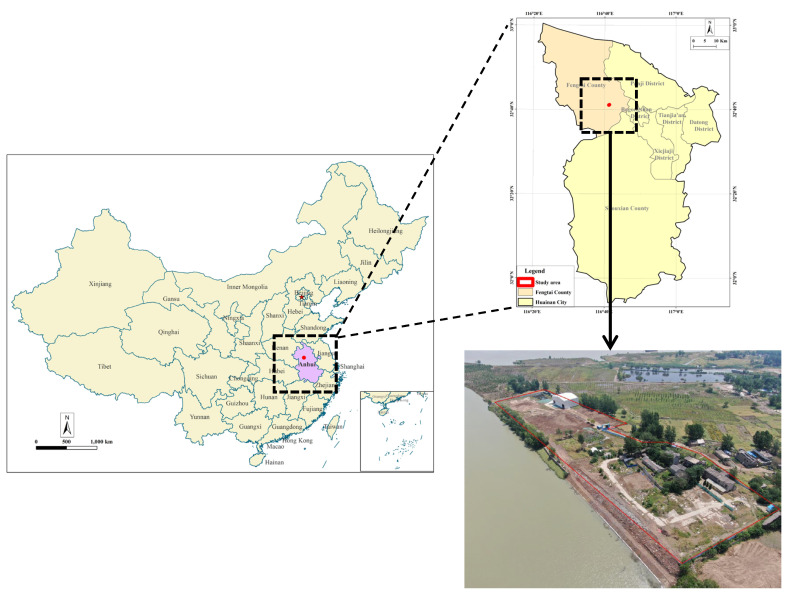
Map of solid waste investigation area.

The study focuses on two decommissioned chemical industry sites that are adjacent to each other, with respective areas of 6860 m^2^ and 2094 m^2^ (Figure 1). The two sites shared similar production products and processes, mainly producing phenyl chlorosilanes and methyltriethoxysilane. The terrain of the sites is relatively flat, located in the Huaihe River first-level terrace geomorphic unit. The upper layers consist of Quaternary artificial fill, Quaternary Upper Pleistocene (Q3^al^) clay, silty clay, Quaternary Middle Pleistocene (Q2^al^) clay, silty clay, and silt [16]. Chemical wastes were landfilled on these sites, which have since been cleared and handled. However, during soil pollution remediation processes, remnants of solid waste or scattered waste were discovered, prompting a comprehensive and thorough investigation of the waste on these sites.

## 3. Methodology

This study utilized a combination of various geophysical detection methods and validation drilling to investigate the solid waste landfill range on the site. By using EVS (v2020.5.2, Gainsville, FL, USA), the drilling results were visualized, along with the ground elevation and solid waste landfill layer markers. A spatial distribution map of the solid waste landfill was created to obtain information on its location and volume. This visualization process serves as a basis for identifying the hazardous characteristics of solid waste, as well as for clean-up and disposal efforts.

### 3.1. Geophysical Detection

Among the geophysical electrical (magnetic) methods, the transient electromagnetic method is a non-destructive, fast, and mobile observation method that uses artificial excitation of electromagnetic fields and then receives induced electromagnetic fields from the target for detection. It is widely used in various detection scenarios, including urban engineering detection and hydrological surveys. The high-density electrical resistivity method achieves profiling and depth sounding by utilizing multi-electrode configurations, one-off electrode deployment, and modifying electrode spacing to observe and measure electrical potential differences. These three methods are suitable for near-surface detection, featuring simple equipment, flexible construction, and lower costs, making them ideal for detailed near-surface investigations. Therefore, this study utilized a comprehensive geophysical electromagnetic detection approach with a focus on the transient electromagnetic method and ground-penetrating radar, which was complemented by the high-density electrical resistivity method.

#### 3.1.1. Transient Electromagnetic Method

The transient electromagnetic (TEM) method employs ungrounded loops to transmit pulsed electromagnetic fields into the subsurface. Receiver coils then measure spatiotemporal distributions of secondary fields induced by subsurface eddy currents. This method is used to address geological issues related to time-domain electromagnetic detection.

#### 3.1.2. High-Density Electrical Resistivity Method

High-density resistivity measurements leverage contrasts between the pollutant and background resistivity. The apparent resistivity is calculated by injecting a current and measuring the inter-electrode potential differences. By collecting a large amount of data and using inversion models, the distribution characteristics of the apparent resistivity of the target on a certain profile can be inferred.

The working mechanism of the high-density electrical resistivity method is as follows: direct current (or ultra-low-frequency current) is supplied to the underground via source electrodes (A, B), while a potential difference (ΔUmn) is observed between measurement electrodes (M, N) and the apparent resistivity (ρs) is calculated. The electrodes are simultaneously or sequentially moved along selected survey lines at specified electrode-spacing intervals. Electrodes are pre-installed manually, with the instrument automatically switching between them. The observation work is simple, efficient, and informative, leading to improved survey capabilities. Data processing for the high-density electrical resistivity method is automatically conducted by the program, ensuring accurate and convenient interpretation. First, a few anomalous points should be smoothed, followed by terrain corrections. Two-dimensional inversion calculations should be performed using the least squares method to produce resistivity inversion results. Interpretation personnel should then make inferences based on anomaly shapes and developmental features to determine layer thickness, infer the geological and physical characteristics of the target substance, and determine its occurrence state, ultimately solving engineering geological problems.

#### 3.1.3. Ground-Penetrating Radar Method

The ground-penetrating radar method utilizes a geological radar-transmitting antenna to emit high-frequency pulsed electromagnetic waves towards the target and receives the reflected electromagnetic waves from the target using a receiving antenna. This method is a geophysical detection technique used to detect the spatial position and distribution of the target, relying on the reflection characteristics of electromagnetic waves from the target and surrounding media to investigate the internal structure and defects (or other inhomogeneities) within the target.

Ground-penetrating radar obtains two-dimensional vertical images of profiles by scanning the entire cross-section of concealed targets with radar antennas. The working mechanism involves the radar system transmitting wide-band high-frequency electromagnetic waves to the underground using an antenna. When the electromagnetic signal encounters a medium interface with a significant dielectric difference during propagation through the medium, reflection, transmission, and refraction occur. The greater the difference in dielectric constants between the two media, the higher the energy of the reflected electromagnetic waves. The motion characteristics of reflected electromagnetic waves are meticulously recorded by the radar host after they are received by the synchronized receiving antenna that moves in tandem with the transmitting antenna. Through signal processing, a complete cross-sectional scan image is generated. Engineering technicians interpret the radar image to determine the actual underground structure of the target. Ground-penetrating radar mainly uses wideband high-frequency time-domain electromagnetic pulse waves for target detection via reflection. By using the formula based on the measured radar wave travel time, the target object’s depth (z) is calculated.

#### 3.1.4. Survey Line Layout

**Figure 2:**
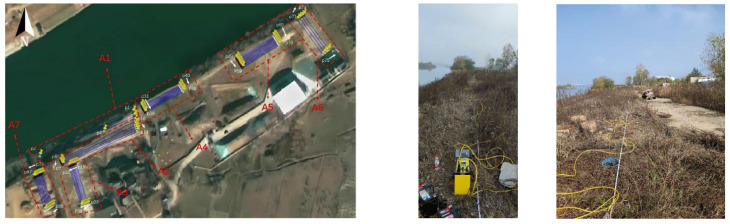
Survey lines layout.

In this study, a total of 7 main areas within the investigation area were detected (Figure 2). The transient electromagnetic method was used for 50 profiles, totaling 2936 physical points measured. Specifically, there were 2 experimental detection profiles, with a total of 120 checkpoints, 48 profiles with 2761 measurement points, and 55 solid waste response experimental measurement points. Two high-density detection profiles were completed, with 192 physical points measured. Furthermore, 22 profiles were detected using the ground-penetrating radar method, with a total length of 1072.1 m.

#### 3.1.5. Data Processing

1. Transient electromagnetic method data processing

**Figure 3:**
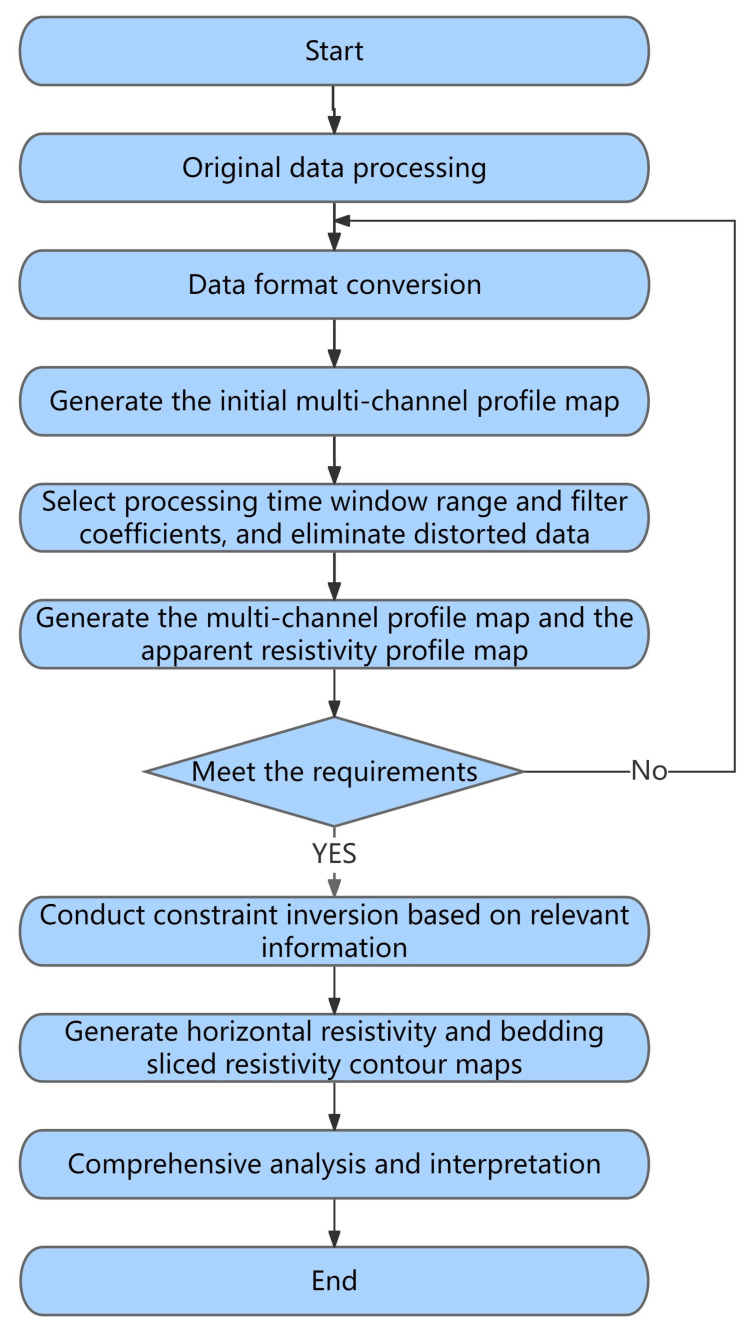
Data processing through transient electromagnetic method.

The data processing for the transient electromagnetic method involves preprocessing the field-measured data; conducting inversion processing on the preprocessed data; and finally, obtaining the apparent resistivity cross-sectional image and contour map [11]. Figure 3 depicts the data processing process using the transient electromagnetic method.

First, it is necessary to correct the disturbed or significantly changing data. Due to the influence of surface metal pipelines, construction sites, geological noise, and other factors, interference anomalies can occur, resulting in extreme spikes or dips in values, which can even distort the overall data. To obtain accurate results, the original data needs to be preprocessed to eliminate interference anomalies, preventing the presence of false anomalies caused by interference. The processing methods include removing extreme values, applying wavelet denoising and smoothing filters within a reasonable range, and then plotting the processed data to provide a reliable basis for subsequent interpretation and analysis.

2. High-density electrical resistivity method data processing

The original data from the high-density electrical resistivity method is imported into the RES2DINV software (v4.8.10, Zurich, Switzerland). The “Eliminate Spurious Measurement Points” function is then used. In this function, the apparent resistivity data is displayed in the form of contours for each measurement layer, allowing for rectification of notably erroneous resistivity values by using a mouse. The RES2DINV software utilizes a smooth-constrained Gauss–Newton least-squares inversion technique to invert underground apparent resistivity structures into a two-dimensional model based on resistivity survey data. This software is widely used for constructing structural profile models of moderately complex geological areas where insufficient one-dimensional resistivity detection information is available.

3. Ground-penetrating radar method data processing

The radar-detected data is recorded as pulse reflection waveforms and displayed as a waveform or grayscale to depict the vertical profiles obtained by the ground-penetrating radar. The interpretation of ground-penetrating-radar-detected data consists of two main parts: data processing and image interpretation.

Data processing involves eliminating random noise, suppressing interference, and improving the background. This includes applying automatic time-variable gain or controlled gain to compensate for medium absorption and suppress noise, filtering to remove high frequencies, highlighting target features, and reducing background noise and ringing effects.

Data processing is carried out using EKKO Project software (V6 R2.1 build 8238, Canada) provided with the instrument. The steps are as follows: (1) subtract the DC shift, (2) move the start time, (3) calculate the gain/energy decay, (4) subtract the average, (5) use a Butterworth bandpass, and (6) calculate the running average.

#### 3.1.6. Data Quality Assessment

1. Transient electromagnetic method data quality assessment

To ensure the data quality of this comparative experiment, the quality inspection was designed to cover no less than 3% of the actual completed task volume. Additionally, the inspection accuracy was designed not to exceed 10%. After the calculations, it was determined that the accuracy requirements for this measurement work were met. The statistical calculation of the actual inspection accuracy was performed according to the following formula:

$$m\prime =\pm \sqrt{\frac{1}{2n}\sum_{i=1}^{n}{\left(\frac{V_{i}-Vi^{\prime }}{\bar{V_{i}}}\right)}^{2}}\times 100$$

where $\bar{V_{i}}==\left(V_{i}+V\prime_{i}\right)/2$ represents the mean square relative error (%) of the quality inspection line, *i* represents the number of measurement points (i = 1, 2… n), $V_{i}$ represents the original observation value of the *ith* measurement point, $V\prime_{i}$ represents the inspection observation value of the *ith* measurement point, and $\bar{V_{i}}$ represents the average observation value of the original and inspection data for the *ith* measurement point.

2. High-density electrical resistivity method data quality assessment

The high-density electrical resistivity method utilizes AGI hardware equipment for data collection. The instrument comes with a “self-check” function, which allows for direct quality inspection of the collected data. If any data anomalies occur during the collection process, adjustments can be made and the data can be rechecked to ensure that the data quality meets the requirements.

### 3.2. Borehole Layout

**Figure 4:**
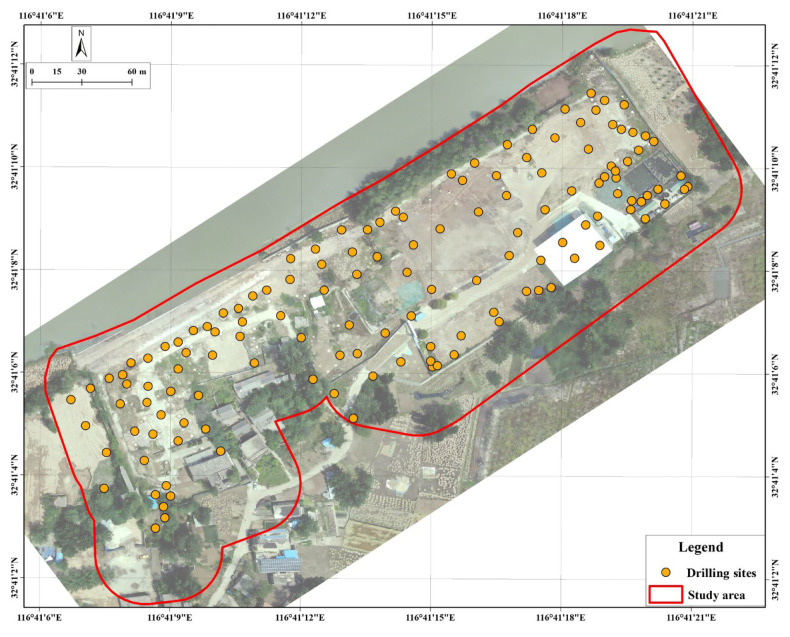
Distribution of drilling points.

According to the systematic borehole layout method, borehole locations were arranged within the investigation area, with a density not less than 10 m × 10 m. A total of 136 borehole locations were set up (Figure 4). Environmentally friendly Geoprobe drilling equipment was used for core sampling, with a maximum drilling depth of 50 cm below the original soil layer at the bottom of the landfill. After drilling was completed, the boreholes were sealed to prevent pollution spread.

### 3.3. Mutual Verification of Geophysical and Drilling Detection Results

For suspected solid waste distribution identified by geophysical detection, Geoprobe drilling equipment was used to conduct drilling verification in the suspected solid waste landfill area. Based on the core profile, the presence of solid waste landfill in the suspected area discovered by geophysical exploration could be determined. If present, additional drilling locations were densified in this area to clarify the extent and depth of the solid waste landfill. For solid waste discovered by drilling equipment, additional drilling locations were densely arranged around the original drilling site to determine the extent of the solid waste landfill.

### 3.4. Earth Volumetric Studio (EVS)

This study utilized EVS (visualization geological modeling software) to conduct spatial interpolation and produce a visual display of the borehole data obtained from the investigation (such as borehole locations and landfill depths). By analyzing the distribution of core samples from the borehole locations, data of the landfill layers were obtained. The entire investigation area was simplified and divided into three geological layers from top to bottom: soil–landfilled waste–soil. Ultimately, information on the spatial distribution of the solid waste landfill and volume of the landfill was obtained.

## 4. Results

### 4.1. Analysis of Geophysical Data

#### 4.1.1. Analysis of Transient Electromagnetic Detection Results

In the 50 transient electromagnetic investigation profiles in seven investigation areas, three investigation areas (A2, A4, A6) were selected for comprehensive three-dimensional imaging slice analyses.

**Figure 5:**
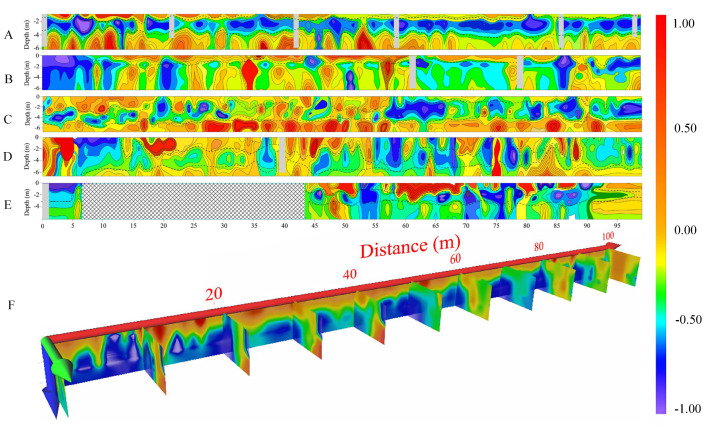
A2 investigation area imaging profile (Due to geological or other factors, the gray and grid parts in the figure represent areas where no observation data have been obtained. A: survey line imaging profile of A2L1, B: Survey line imaging profile of A2L2, C: Survey line imaging profile of A2L3, D: Survey line imaging profile of A2L4, E: Survey line imaging profile of A2L5, F: Three-dimensional imaging slices of the A2 investigation area).

A2 shows a northeast–southwest trend overall, with a length of approximately 100 m and a width of about 10 m. A total of five survey lines were arranged, named A2L1 to A2L5, with a spacing of 1 m. The resistivity profile of A2 vertically exhibits a “high-low” distribution trend, and the overall electrical structure is relatively continuous (Figure 5). Based on the electrical characteristics, the geological layers in A2 can be broadly divided into two layers: the surface layer consists of a fill soil layer containing construction solid waste, gravel, and surface vegetation roots, with relatively low water saturation and relatively high resistivity; the second layer is a clay layer with relatively high water saturation and low resistivity. According to the imaging profiles of each survey line (Figure 5), A2L1 to A2L3 may indicate the presence of solid waste landfill. The A2L1 profile shows strong layering characteristics in the dielectric medium. The 0–2 m depth range contains the surface fill soil layer, while the 2–4 m depth range is the clay layer with relatively low resistivity. This profile result is well-correlated with the layer division results obtained from the A2L1 core drilling. With drilling and geological data, the area where solid waste may exist is likely to be within the region of moderate resistivity in the low-resistivity clay layer. In the initial 0 to 5 m of the A2L2 profile, there is a large low-resistivity area, which does not show clear correlations with A2L1 and A2L3. Therefore, it was inferred that this low-resistivity area may be due to the localized presence of metal or excessive water saturation. Solid waste in A2L2 may be landfilled at a depth of around 2 m, with possible solid waste landfill at 60 and 90 m on the profile. The A2L3 profile is generally divided into three layers, with the fill soil layer within 2 m showing generally elevated resistivity, but from 57 to 90 m, the fill soil layer becomes thinner, possibly due to increased water saturation causing a decrease in resistivity. The middle layer shows heterogeneous electrical properties with significant lateral variations. Based on the characteristics observed in the A2L1 profile, it was inferred that the A2L3 profile between 66 and 85 m may contain solid waste landfill.

**Figure 6:**
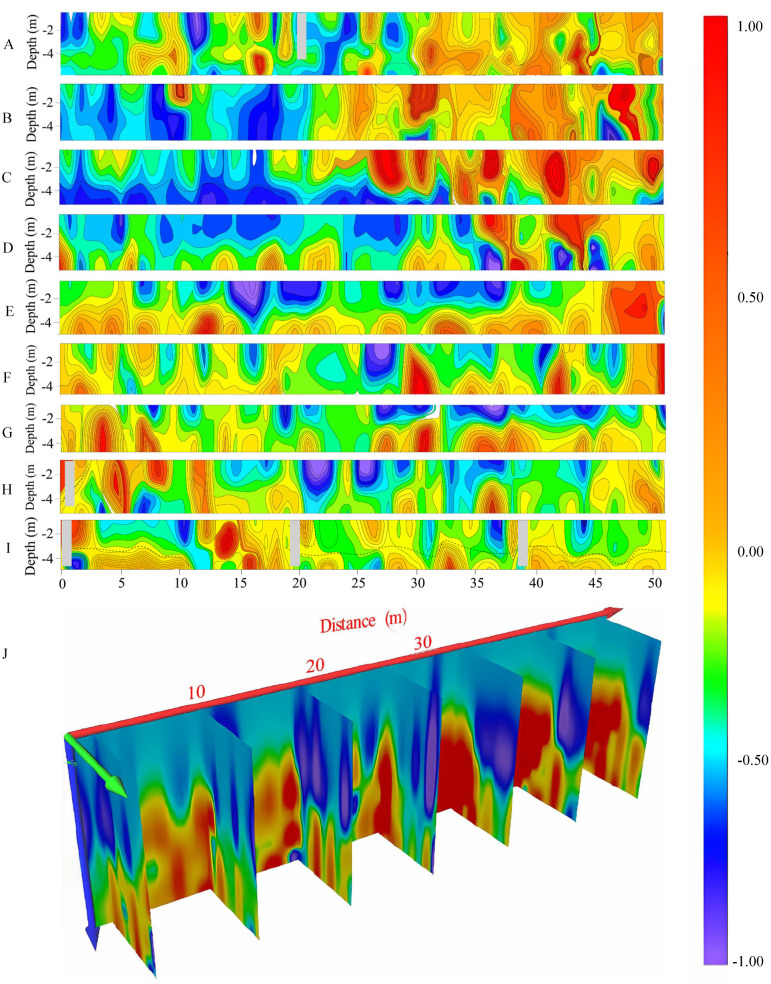
A4 investigation area imaging profile (A: survey line imaging profile of A4L1, B: survey line imaging profile of A4L2, C: survey line imaging profile of A4L3, D: survey line imaging profile of A4L4, E: survey line imaging profile of A4L5, F: survey line imaging profile of A4L6, G: survey line imaging profile of A4L7, H: survey line imaging profile of A4L8, I: survey line imaging profile of A4L9, J: three-dimensional imaging slices of the A4 investigation area).

A4 shows an overall northeast–southwest trend, with a length of approximately 52 m and a width of around 8 m. A total of nine survey lines were arranged, labeled as A4L1 to A4L9, with a spacing of 1 m between points and lines. Figure 6 shows that the resistivity in A4 gradually increases with increasing depth, with good uniformity in resistivity in the shallow layers and relatively low resistivity in the geological layers. The middle-to-deep layers show poor lateral uniformity, with the northeastern region of the investigation area exhibiting high resistivity, the southeastern region showing low resistivity, the western area predominantly comprising medium-resistivity layers, and some areas exhibiting low-resistivity anomalies. Based on the imaging profiles of each survey line (Figure 6), A4L3 and A4L9 may indicate the presence of solid waste landfill. The A4L3 profile shows a clear layered structure, with relatively high resistivity in the overlying layer and relatively low resistivity in the underlying layer, and the resistivity distribution is relatively uniform. However, there are significant high-resistivity anomalies in the centers of the 28 to 31 m and 34 to 37 m sections of the profile, indicating a potential presence of solid waste landfill in these areas. The A4L9 profile can be divided into two layers based on electrical differences in the geological layers, with a continuous high-resistivity anomaly in the 13 to 16 m section, suggesting the possible presence of solid waste landfill.

**Figure 7:**
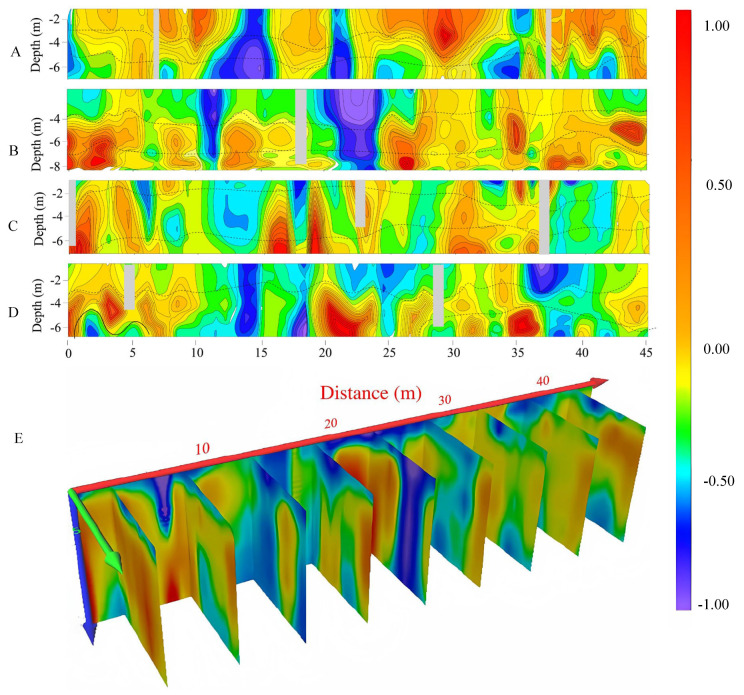
A6 investigation area imaging profile (A: survey line imaging profile of A6L1, B: survey line imaging profile of A6L2, C: survey line imaging profile of A6L3, D: survey line imaging profile of A6L4, E: three-dimensional imaging slices of the A6 investigation area).

A6 shows an overall northwest–southeast trend, with a length of approximately 46 m and a width of around 3 m. A total of four survey lines were arranged, labeled as A6L1 to A6L4, with a spacing of 1 m between lines and points. The resistivity in the A6 investigation area gradually increases from shallow to deep layers, with good uniformity in resistivity in the shallow layers and relatively low amplitudes. The middle-to-deep layers mainly consist of medium-to-high-resistivity geological layers (Figure 7). The drilling core results within A6 show that there are some contaminated layers at depths between 4 and 5 m. The thickness of the contaminated layer ranges from 0.2 to 1 m and mainly comprises black clay, which was possibly formed by the infiltration of chemical waste liquid mixing with the original basement layers and soil.

#### 4.1.2. Analysis of High-Density Electrical Resistivity Detection Results

**Figure 8:**
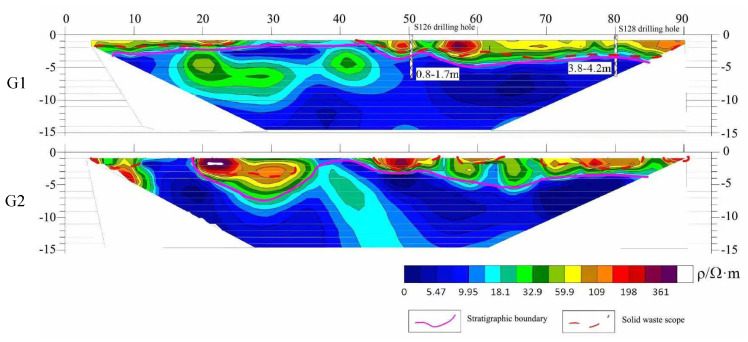
Resistivity inversion profiles of G1 and G2 using high-density electrical resistivity method.

The primary objective of the high-density electrical resistivity method in this study was to identify the spatial distribution of solid waste, complementing and mutually verifying the transient electromagnetic and ground-penetrating radar methods. Two profiles (G1 and G2 lines) for high-density electrical resistivity detection were arranged within A2. Figure 8 shows the two-dimensional inverted resistivity profiles of G1 and G2; their overall electrical distributions exhibit similarities and correlate well with the profiles obtained from the transient electromagnetic detection. Boreholes S126 and S128 are located at 50 and 80 m along G1, respectively, revealing pollutant depths of 0.8 to 1.7 m (with a thickness of 0.9 m) and 3.8 to 4.2 m (with a thickness of 0.4 m). Based on this information, the interpretation of the high-density electrical resistivity detection results for G2 was conducted. Due to the volume effect of the direct current method, the interpreted thickness of the solid waste is greater than the actual thickness. Below 5 m, the geological layers’ physical properties are relatively homogeneous, suggesting undisturbed original strata.

#### 4.1.3. Analysis of Ground-Penetrating Radar Detection Results

**Figure 9:**
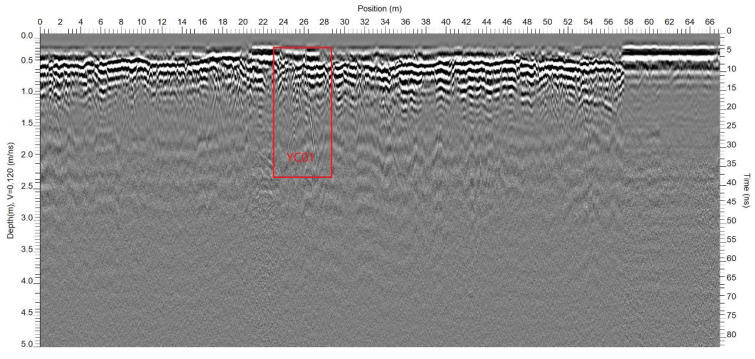
A2-1 GPR profile.

The main objective of the ground-penetrating radar (GPR) method in this study was to aid in the delineation of shallow geological structures (primarily within a 5 m depth), complementing and mutually verifying the transient electromagnetic and high-density electrical resistivity methods (Figure 9). A total of 22 GPR profiles were set up for this study, identifying a total of 17 GPR anomalies (Table 1). Among them, one anomaly exhibits strong reflection signals (Figure 9), displaying a distinctive phase characteristic of an isolated body, which manifested as regular hyperbolic waveform features and a noticeable instantaneous phase, frequency, and amplitude. The initial reflected wave is negative, and strong reflection interface signals persist below it, with a substantial time lag between the two signal sets. Through on-site inspection and analysis, it is conjectured that this anomaly is caused by landfilled solid waste. Other anomalies are thought to be caused by localized voids in the subsurface media, non-compactness, and the presence of construction debris, among others.

### 4.2. Analysis of Drilling Results

**Figure 10:**
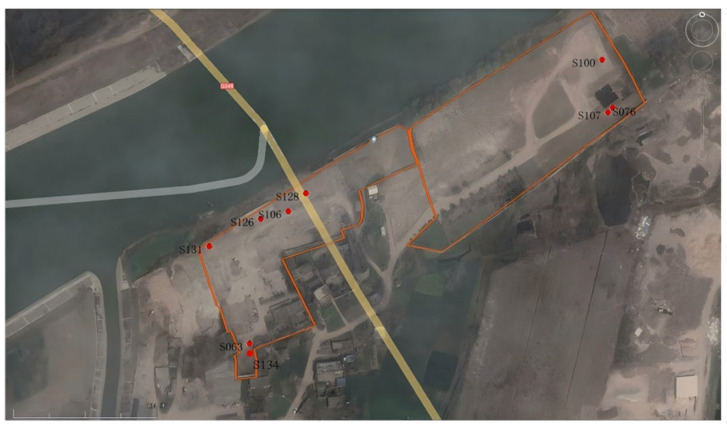
Distribution of solid waste landfill sites (S106, S126, S128, S131: construction debris, coal ash, and contaminated soil mixture; S63, S134: hazardous distillation waste; S76, S100, S107: household waste).

Through the sampling work conducted at 136 drilling locations on the site, based on comprehensive assessments of the core profile, color, odor, etc., a total of nine locations with solid waste landfill were identified. These locations are concentrated in three areas (see distribution in Figure 10), each exhibiting different characteristics of solid waste. One location contains construction debris, coal ash, and contaminated soil mixture; another location has black crystalline solid waste with a pungent odor, preliminarily identified as hazardous distillation waste residue from the production process; and the third location consists of household waste, appearing black with a slightly unusual odor.

### 4.3. Interpolation Simulation of the Three-Dimensional Spatial Distribution of Landfilled Waste

**Figure 11:**
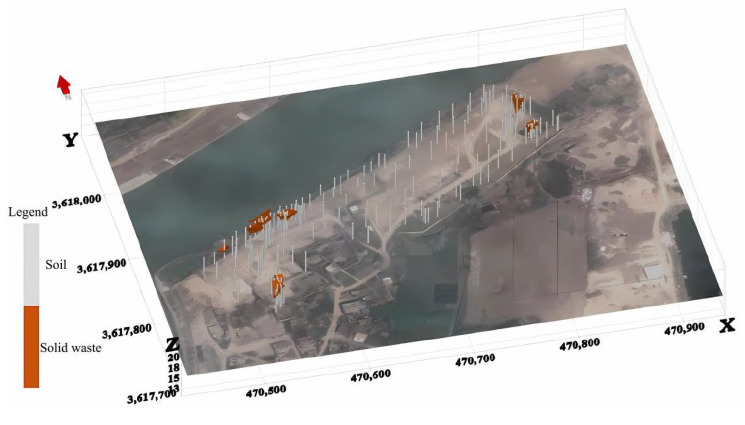
Simulation of three-dimensional spatial distribution of landfilled waste.

This study utilized EVS2020 for spatial interpolation and visual representation of the borehole data obtained from the site investigation. By utilizing 54 drilling locations within the site, data on the layers with landfilled waste were obtained, and the geological layers of the entire site were briefly divided from top to bottom into three layers: soil–landfilled waste–soil. Due to the spatial distance between the three landfill areas within the site, the study involved zoning interpolation of the areas containing landfilled waste based on the drilling results. By using EVS, the spatial distribution of the landfilled waste within each zone was simulated, with the Kriging interpolation method applied for grid interpolation. The simulation results (shown in Figure 11 with the Z-axis scaled up by a factor of 10) indicate that the total volume of solid waste landfilled within the site is approximately 266.9 m^3^.

## 5. Discussion

Our integrated approach combining transient electromagnetic (TEM), high-density resistivity, ground-penetrating radar, and validation drilling methods systematically characterized 3D solid waste distribution. The TEM and borehole data show strong consistency (e.g., 0–4 m electrical layering in A2L1 matched the core horizons), confirming that contrasts in electrical properties enable reliable layer differentiation [20]. Notably, waste bodies produced diagnostic medium–high resistivity anomalies (100–300 Ω·m) in the A2/A4 zones, aligning with established response patterns [21]. Conversely, localized low-resistivity zones (<50 Ω·m) indicate metallic/high-moisture interference, underscoring the need for geological contextualization [22].

Critically, the quantified landfill volume of 266.9 m^3^, though spatially fragmented, exceeds the threshold (e.g., >100 m^3^) requiring mandatory remediation under China’s Technical Guidelines for Risk Assessment of Soil Contamination of Land for Construction (GB 36600-2018) [23] for industrial sites. Furthermore, through multi-method cross-validation discovery, the high-density electrical method in A2 successfully reproduced the TEM stratification structure, but its overestimation of waste thickness caused by volumetric effects (Figure 9) confirms the vertical resolution limitations of DC electrical methods indicated in [24,25,26]. Meanwhile, ground-penetrating radar’s precise capture of shallow anomalies (e.g., YC01 hyperbolic feature) demonstrates the geometric body reflection wave phase identification model established in [27,28,29]; among its delineated 17 anomalies, only 1 was confirmed as solid waste, profoundly revealing the non-uniqueness essence of geophysical interpretation. This challenge highly coincides with practical experience in the Yangtze River Delta contaminated site investigation, highlighting the irreplaceability of borehole verification [30].

Borehole data further indicate that the solid waste spatial distribution presents significant sporadic fragmentation characteristics (Figure 11), with the landfill thickness being only 0.2–1.3 m with no vertical pattern; combined with site historical cleanup records, this can be attributed to residual effects from prior remediation projects. This phenomenon matches the typical pattern of “discrete contamination patches formed after partial cleanup of informal landfills” reported [31], while the thin-layer contaminated clay (0.2–1 m thickness) in A6, though limited in scale, contains organic pollutants (phenyl chlorosilanes, methyltriethoxysilane) classified as hazardous waste under China’s National Catalogue of Hazardous Wastes (HW13). Its resistivity signatures (medium–high anomalies) correlate with confirmed organic contamination thresholds. As such, this study innovatively adopted a zonal Kriging interpolation strategy (EVS2020) to construct a solid waste 3D model; through reducing the spatial heterogeneity error, we successfully quantified the total landfill volume as 266.9 m^3^. This method and the “heterogeneous stratum partitioning optimization algorithm” proposed in [32] lead to technical consistency; however, they are limited by the borehole density (54 points) and the model’s characterization capability for thin-layer solid waste (<0.5 m) is still constrained by the “Kriging smoothing effect under sparse data” described in. Future work could integrate geophysical–borehole joint modeling technology developed to overcome these issues.

The detection of thin waste layers (0.2–1.3 m) revealed critical resolution constraints: TEM’s limited vertical resolution (>3 m at a 5 m depth) missed 60% of the <0.5 m layers in the A6 clay, causing a ~15% volume underestimation; GPR’s 100 MHz antennas achieved a 0.3 m resolution, but conductive clay (εr > 25) reduced it to 0.5 m, yielding only 1 waste-confirmed anomaly in 17 detections; and HDR’s 1 m electrode spacing inflated the thickness measurements by 50–200% (e.g., 0.4 m waste imaged as 0.9–1.2 m). These limitations, compounded by near-surface heterogeneity (e.g., metallic debris generating false positives), necessitate adaptive drilling densities; we recommend 15% more boreholes in complex zones than in theoretical grids.

The transferability of the interpretation model is context-dependent for several reasons: (1) Resistivity thresholds (100–300 Ω·m) are valid for Huaihe Terrace’s clay–silt sequences but drop to <50 Ω·m in coastal saline sediments, requiring pre-site electromagnetic noise mapping. (2) The waste composition critically affects signatures: >5% metallic content inverts medium–high to low resistivity, as observed in electronics waste sites. (3) Lastly, scale effects demand a 3× geophysical line density for landfills >50,000 m^2^. Validation across three Jiangsu sites showed 22–41% resistivity variation for identical waste, underscoring the need for machine learning compensation and a national waste property database to standardize cross-regional assessments.

Regarding industry practice, this study’s integrated detection system has significant methodological value: TEM’s effectiveness for deep structures (e.g., medium–high-resistivity layer below 5 m in A6) and ground-penetrating radar’s effectiveness for shallow targets (within 5 m) are complementary and can cover a contaminated site’s 0–15 m critical depth range; the high-density electrical method’s volumetric effect warning reinforces the quantitative geophysical borehole calibration principle required standard]; and the identification of hazardous organic residues and quantified waste volume providess actionable data for implementing risk control levels (e.g., intervention values for benzene homologs ≤1 mg/kg in GB 36600-2018), supporting regulatory decision-making for site remediation.

## 6. Conclusions

(1) The geophysical data highlight two main characteristics of the anomaly distribution: first, anomalies exhibit a “sparse distribution with multiple points”; second, there is a variety of anomalies, including solid waste anomalies, remnants of the original factory building foundations, contaminated soil, voids, cracks, and household waste. These types were differentiated by synthesizing multiple data types, including geophysical responses and historical records (personnel interviews), and by conducting drilling verification. The anomalous areas are mainly distributed in the A2, A4, and A6 investigation areas, with solid waste primarily located in A2, featuring “multiple points, small quantity”. A4 may contain a small amount of suspected landfilled solid waste, while the anomalies in A6 are mainly caused by the contaminated soil layers, which are interspersed with landfilled garbage according to personnel interviews and drilling data.
(2) A total of 136 drilling locations were investigated within the site, identifying nine drilling locations with solid waste landfill that are concentrated in three specific areas. The characteristics of the solid waste vary, and their vertical distribution is irregular. The landfill thickness ranges from 0.2 to 1.3 m. This suggests that incomplete clearance could be the reason for the persistence of residual solid waste and their irregular distribution and sporadic patterns.
(3) Based on the drilling data (location distribution, landfill thickness, etc.), EVS was utilized to conduct a spatial distribution simulation of the landfilled waste using the Kriging interpolation method. The simulation estimated that the total volume of solid waste landfilled within the site is 266.9 m^3^.
(4) Integrating targeted drilling with geophysics resolved the accuracy limitations, providing high-resolution spatial waste distribution data. This strategy reduces investigation costs by >30% and establishes a replicable framework for landfill characterization.

## Figures and Tables

**Table 1:** Statistics of GPR anomalies.

Line No.	Anomaly No.	Anomaly Position (Horizontal)	Affected Depth	Inference and Interpretation
A2-1	YC01	22.8~28.8 m	0.3~2.4 m	Solid waste
A3-1	YC02	17.6~23 m	0.6~1.8 m	Isolated stone
	YC03	32.5~37.2 m	0.6~1.8 m	Non-compactness
	YC04	39~43.2 m	0.5~1.2 m	Isolated stone
A3-2	YC05	15.4~16.8 m	0.5~2.2 m	Localized void
	YC06	18.5~20.8 m	0.75~1.9 m	Localized void
	YC07	41.5~44 m	0.75~1.2 m	Localized void
A3-3	YC08	16.5~19.5 m	0.4~1.4 m	Localized void
A3-4	YC09	13~16 m	0.4~0.8 m	Non-compactness
A3-5	YC10	4.2~7.4 m	0.3~1.1 m	Cavity
A5-2	YC11	30.5~33.6 m	0.8~1.7 m	Construction debris
A5-6	YC12	39~44 m	1.7~2.4 m	Non-compactness
A5-7	YC13	39~43.8 m	1.7~2.5 m	Non-compactness
A6-1	YC14	10.8~13.6 m	0.6~1.4 m	Localized void
	YC15	17.8~21.8 m	1~1.9 m	Localized void
A6-2	YC16	8.5~11.8 m	0.6~2 m	Non-compactness
	YC17	14.3~17 m	0.5~2.3 m	Non-compactness

## Data Availability

The original contributions presented in this study are included in the article; further inquiries can be directed to the corresponding authors.
